# Respiratory Syncytial Virus: A Systematic Review and Meta-Analysis of Tomographic Findings (2000–2022)

**DOI:** 10.3390/children10071169

**Published:** 2023-07-05

**Authors:** Matteo Riccò, Silvia Corrado, Sara Palmieri, Federico Marchesi

**Affiliations:** 1 Local Health Unit of Reggio Emilia, Servizio di Prevenzione e Sicurezza Negli Ambienti di Lavoro (SPSAL), AUSL–IRCCS di Reggio Emilia, 42122 Reggio Emilia, Italy; 2 UOC Pediatria, Dipartimento della Donna e Area Materno-Infantile, ASST Rhodense, 20024 Garbagnate Milanese, Italy; 3 Dipartimento Diagnostica per Immagini, ASST Spedali Civili di Brescia, Radiologia 1, 25123 Brescia, Italy; 4 Department of Medicine and Surgery, University of Parma, 43126 Parma, Italy

**Keywords:** computed tomography, RSV, viral pneumonia, differential diagnosis

## Abstract

Human respiratory syncytial virus (RSV) is a main cause of medical referrals and hospitalizations in all infants, particularly among newborns. Nevertheless, relatively limited evidence on chest tomography (CT) findings has been collected. According to the PRISMA statement, Pubmed, Embase, and medRxiv were searched for eligible observational studies published up to 31 December 2022. Cases were categorized in children and adolescents (age < 18 years), adults and elderly (age ≥ 18 years), and immunocompromised patients, and then pooled in a random-effects model. Heterogeneity was assessed using the I2 statistics, while reporting bias was assessed by means of funnel plots and regression analysis. A total of 10 studies (217 RSV cases) were retrieved (children, 37.3%; immunocompromised, 41.0%; adults, 21.7%). The most common features were signs of organizing pneumonia (33.65%, 95% confidence interval [95% CI] 22.39–47.27), followed by septal thickening (33.19%, 95% CI 21.76–47.03), ground glass opacities (GGOs; 28.03%, 95% CI 14.69–46.82), and tree-in-bud (TIB, 27.44%, 95% CI 15.04–44.68). Interestingly, up to 16.23% (95% CI 8.17–29.69) showed normal findings, while the large majority (76.06%, 95% CI 64.81–84.56) were characterized by bilateral involvement. Studies were highly heterogeneous without substantial reporting bias. Assuming children and adolescents as reference groups, healthy adults were characterized by a higher risk ratio [RR] for septal thickening (RR 3.878, 95% CI 1.253–12.000), nodular lesions (RR 20.197, 95% CI 1.286–317.082), and GGOs (RR 2.121, 95% CI 1.121–4.013). RSV cases are rarely assessed in terms of CT characteristics. Our study identified some specificities, suggesting that RSV infections evolve heterogeneous CT features in children/adolescents and adults, but the paucity of studies recommends a cautious appraisal.

## 1. Introduction 

Human respiratory syncytial virus (RSV) is a respiratory viral pathogen belonging to the genus Orthopneumovirus (family Pneumoviridae) [1,2,3]. With a total of 33 million cases of acute lower respiratory tract infections (i.e., bronchiolitis and pneumonia; LRTI), RSV infections are acknowledged as a quite common cause of morbidity among young children [3]. Nonetheless, an even greater burden of disease has been suspected, as nearly all children become infected with RSV by 2 years of age, most of them only developing mild respiratory symptoms [1,2,3], with a well-defined seasonal trend [2,4]. Even though around 90% of incident cases are usually managed as outpatients [3], RSV infections cause high hospitalization rates, even in otherwise healthy infants [5,6,7,8]. In this regard, it should also be stressed that RSV is not limited to pediatric-age subjects [9,10], but can cause a substantial burden of disease in adults, more precisely among immunocompromised individuals [11,12] and the elderly [12,13], particularly among institutionalized subjects [12,14]. As a consequence, RSV has a substantial global impact, in terms of direct and indirect costs [15,16,17,18,19,20].

Even though a preventive vaccine has recently been licensed [21,22], its use is limited to the older adults, and the appropriate management of RSV illnesses remains far from optimal [23,24,25,26], particularly in pediatric age groups [23,24,25,26]. In fact, the proper handling of RSV cases, particularly of the most severe ones, is often hindered by the lack of a timely diagnosis [27,28,29]. On the one hand, respiratory syndromes associated with RSV have a variable clinical presentation that can lead to a complicated differential diagnosis from other respiratory infections (e.g., bacterial pneumonia, viral upper respiratory tract infections, acute otitis media, and sinusitis) [27]. On the other hand, obtaining appropriate specimens from the respiratory tract may be quite difficult, particularly in children and small infants, as the collection of lower airway secretions requires invasive diagnostic procedures that cannot be routinely used, particularly in newborns and infants aged less than the 2 years (i.e., the highest risk group) [28,29,30]. Unfortunately, noninvasive procedures for the collection of pulmonary specimens (e.g., exhaled breath condensate) have not been extensively validated, and available studies have stressed their substantial shortcomings [31,32,33,34]. Moreover, the upper respiratory tract may not accurately reflect the cause of the clinical syndrome, while rapid diagnostic tests on nasal, rhinopharyngeal, or salivary specimens usually lack diagnostic accuracy and are of limited value in the managing of differential diagnosis [27,28,29,30,35].

The early stages of the SARS-CoV-2 pandemic have stressed how chest imaging through computed tomography (chest CT) may be quite useful in guaranteeing a rapid differential diagnosis for respiratory infection syndromes [27]. As the majority of respiratory pathogens, including SARS-CoV-2, are characterized by substantial clinical overlap but potentially distinctive imaging manifestations, it is reasonable that techniques such as chest CT may retain their importance as a reliable tool for assisting clinical professionals in the post-pandemic practice [27,36]. Nevertheless, as current guidelines recommend that infants with a suspected diagnosis of bronchiolitis should be evaluated by means of noninvasive options (such as point-of-care ultrasonography), reserving imaging studies only for most severe cases [37,38], relatively limited evidence has been collected on the chest CT findings of RSV [36,39,40,41,42]. Therefore, the aim of this study was to systematically collect and review the chest CT findings of RSV in children compared to other potential groups (i.e., adults, and immunocompromised individuals). Retrieved features were then meta-analyzed in order to assess which ones may more properly assist in providing a timely differential diagnosis of RSV compared to other respiratory viruses.

## 2. Materials and Methods

A systematic review and meta-analysis of the literature was performed according to the “Preferred Reporting Items for Systematic Reviews and Meta-Analysis” (PRISMA) guidelines [43], and registered into the international database of prospectively registered systematic reviews in health and social care, welfare, public health, education, crime, justice, and international development (PROSPERO) with the progressive registration number CRD42022382636 (see Prisma Checklist as Supplementary Materials).

As a preliminary step, research concepts were defined according to the “PICO” strategy (i.e., patient/population/problem; intervention; control/comparator; outcome), as reported in Table 1.

More precisely, the population (P) of interest was identified in children and adults with diagnosis of respiratory infection; the investigated test results (I) were the reporting of any of the following imaging features: bronchitis, alveolar involvement, bronchial wall thickening, ground glass opacities, nodules, tree-in-bud, consolidation, and pleural effusions; the comparator (C) was identified in the features of other viral respiratory infections identified in the same study populations; the inquired outcome (O) was represented by the association of imaging features with RSV infection.

Three databases were searched for relevant studies from inception up to 31 December 2022, without backward chronological restrictions, more precisely, the scholarly databases PubMed/MEDLINE and EMBASE, as well as the preprint repository medrxiv.org. The search strategy resulted from the combination of the following keywords, both as free text and as Medical Subject Heading [MeSH] terms: (“RSV” OR “respiratory syncytial virus” OR “bronchiolitis”) AND (“chest tomography” OR “chest imaging” OR “imaging” OR “tomography”).

All articles written in Italian, English, German, French, or Spanish (i.e., the languages spoken by the investigators) were included in the analyses.

Records were initially handled by means of the references management software (Mendeley Desktop Version 1.19.5, Mendeley Ltd., London, UK, 2019), with the title and abstract screened by two independent authors. Only articles reporting on original results were retained; where a retrieved article reported any data on RSV from previous studies, the original report was obtained and fetched into the systematic review. On the other hand, review articles, meta-analyses, case reports, case series with <5 cases, meeting reports, and conference abstracts were excluded from both qualitative and quantitative analysis. Articles that were consistent with the aims of the study were then text-reviewed in order to assess whether they met the following inclusion criteria:

1. Reporting crude number of assessed cases of respiratory infections;
2. Reporting the number of RSV cases diagnosed;
3. Reporting the number of CT scans actually performed;
4. Any laboratory diagnosis of RSV infections (i.e., polymerase chain reaction [PCR], immunofluorescence, antigen testing, etc.).

Data extracted included the following:

1. Settings of the study;
2. Characteristics of the index cases, whether they were otherwise healthy children and adolescents (age < 18 years), adults (age ≥ 18 years), or individuals affected by any immune deficiency;
3. Number of included respiratory infection cases;
4. Number of total cases assessed;
5. Number of RSV infection episodes;
6. Number of other lower respiratory tract viral infections, particularly influenza, SARS-CoV-2, and all other viral infections.
7. The following main findings [27,36,45,46]: laterality of the involvement (unilateral vs. bilateral); extensive vs. focal involvement; imaging pattern (i.e., ground glass opacities [GGOs]; nodular lesions; signs of Tree-in-Bud; signs of organizing pneumonia; signs of septal thickening; signs of crazy paving); signs of pleural effusions.

We only included data when the parent study reported CT findings through a “per-patient” approach, while “per-lesion” analyses were excluded as potentially misleading [27]. In order to avoid duplication of data, when multiple publications reported on the same study population, only the most comprehensive study was eventually included in the analyses.

After data extraction, studies were rated about the potential risk of bias by means of the second version of the Quality Assessment of Diagnostic Accuracy Studies (QUADAS-2), a tool for systematic reviews of diagnostic accuracy studies [47]. This tool comprises four domains: patient selection, index text, reference standard, and flow and timing. All domains were assessed in terms of risk of bias (ROB). All articles were rated according to the current indications by two investigators who independently read the full-text versions of eligible articles. Disagreements were resolved by consensus between the two reviewers; when it was not possible to reach consensus, input from a third investigator (M.R.) was searched and obtained.

A descriptive analysis was initially performed by calculating crude prevalence figure per 100 cases in terms of RSV infections over the total of sampled cases, and in terms of occurrence of the main findings by the total number of sampled scans. Similar estimates were also calculated for cases associated with the diagnosis of seasonal flu, COVID-19, and all other viral infections as a whole (i.e., non-RSV viral pneumonia). The crude occurrence rates of the main findings were initially compared in terms of sampled patients (i.e., children/adolescents vs. adults and immunocompromised individuals) and viral diagnoses (i.e., RSV cases vs. seasonal flu, COVID-19, all lower respiratory tract viral infections) by calculating of the corresponding crude risk ratios (cRR) with their 95% confidence intervals (95% CIs). In the calculations, the reference groups were identified in the children/adolescents and RSV infections, respectively.

Pooled estimates for the prevalence rates of main findings and corresponding pooled RR (pRR) for their occurrence in viral pathogens compared to RSV were calculated through a meta-analysis that, in order to cope with the presumptive heterogeneity in study design, was designed through a random effect model. The I^2^ statistic was then applied in order to estimate the amount of inconsistency between included studies (i.e., the percentage of total variation across studies that could be associated with underlying heterogeneity rather than chance), and the following categorization was taken in account: I^2^ ranging between 0% and 25% = low heterogeneity; I^2^ ranging between 26% and 50% = moderate heterogeneity; I^2^ ≥ 50% = substantial heterogeneity.

Publication bias was then investigated through calculation of the contour-enhanced funnel plots, and Egger test for quantitative publication bias analysis (at a 5% of significance level). Radial plots were then calculated and visually inspected to rule out small study bias.

All analyses were performed by means of “meta”, “metafor”, and “robvis” packages with R (version 4.0.3) [48] and RStudio (version 1.1.463) software. The aforementioned packages are open-source add-ons for conducting meta-analyses.

## 3. Results

**Figure 1:**
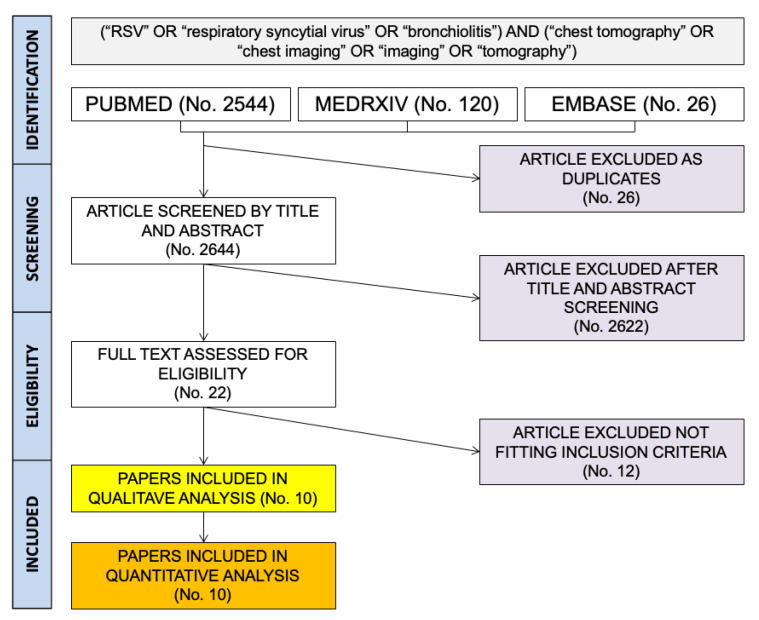
Flowchart of included studies.

As shown in Figure 1, a total pool of 2670 entries (i.e., 2544 from PubMed; 120 from MedRxiv; 26 from EMBASE) were initially retrieved; of them, 26 were duplicated entries and, therefore, removed (No. 2644, 99.0%). The remaining articles were, therefore, screened by title and abstract, with 2622 of them being also removed from the analyses (98.2% of the initial sample).

A total of 22 entries were assessed and reviewed by full text, and 12 of them were excluded as not fitting inclusion criteria [49,50,51]. The remaining 10 papers were eventually included in qualitative and quantitative analysis (0.4% of the initial sample) [41,42,52,53,54,55,56,57,58,59].

A detailed description of individual studies included in qualitative and quantitative analysis is shown in Table 2. Briefly, all retrieved studies were of retrospective design, being performed between 1993 and 2020. A total of 1276 index cases were included, with a sample size ranging between eight cases from the case series of Nabeya et al. [57], and 384 from the study of Ren et al. [54], performed on children/adolescents recruited during the early stages of the SARS-CoV-2 pandemic. Three studies were performed in mainland China, during the first months of the SARS-CoV-2 pandemic [53,54,58], with three further studies from the USA [42,52,59], and one study each from South Korea [55], Japan [57], Germany [56], and Brazil [41].

Regarding the characteristics of the patients, three studies were performed on children/adolescents [53,54,58], along with four on adults [52,55,57,59] and 3 further studies on immune-compromised individuals of various age groups [41,42,56]. While other studies defined pediatric age group as encompassing all individuals younger than 18 at the recruitment, in Kim et al.’s study, a slightly different cutoff for the adult age group was applied (i.e., 16 years) [55].

**Figure A1:**
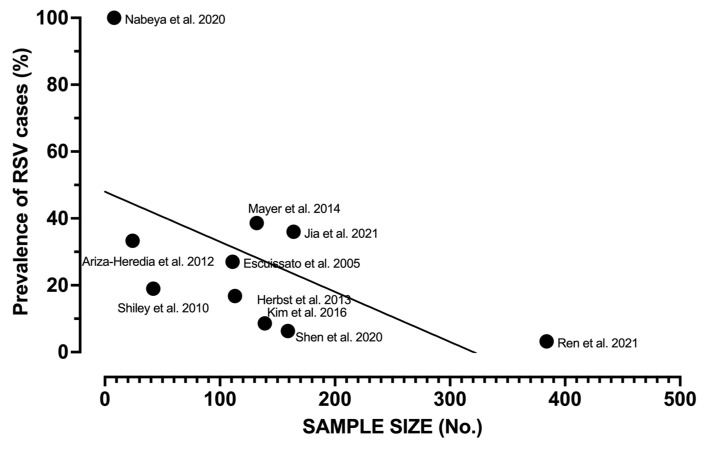
Graphical representation of the correlation between sample size and prevalence of RSV [41,42,50,52,53,54,55,56,57,58]. A nonsignificant negative correlation (rho = −0.564, *p* = 0.096) was identified on Spearman’s rank test.

The reference laboratory study for the diagnosis of RSV was represented by RT-qPCR (alone or associated with antigen testing) in most included studies, while only two reports [41,53] relied on immunofluorescence analysis. A total of 217 RSV cases were eventually included, for a share of RSV cases over the total of sampled individuals of 17.0%, ranging in the individual studies between 3.2% in the report from Ren et al. [54] and 100% in the case series of Nabeya [57]. As shown in Figure A1, the reported share of RSV cases was negatively correlated with the size of the sample (i.e., the smaller the sample, the higher the share of RSV cases) with Spearman’s rho = −0.564, *p* = 0.096.

In seven out of 10 studies, other viral diagnoses were included, either as a general diagnosis of viral pneumonia and by individual pathogens, including seven reports on seasonal influenza, and three reports on SARS-CoV-2, while three reports only included data on RSV cases [42,56,57].

**Figure 2:**
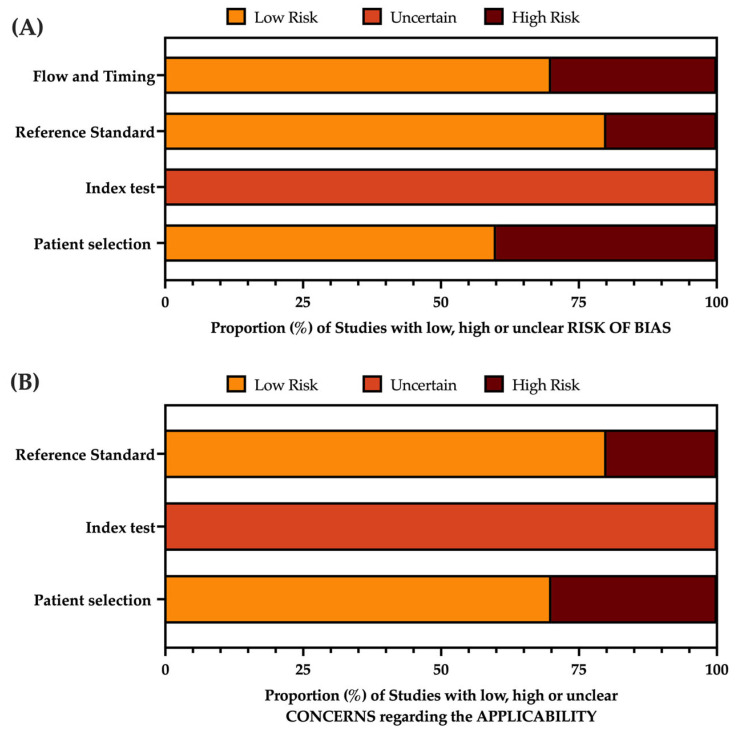
Graphical display of QUADAS-2 results in terms of risk of bias (**A**) and applicability concerns (**B**).

The corresponding ROB assessment is summarized in Table A2 and Figure 2.

Briefly, when dealing with selection bias, fur out of 10 studies were potentially affected to some degree [41,42,56,57]. In fact, studies specifically targeting immunocompromised patients, by their design, could have over-represented milder cases vs. more severe ones because of the perceived frailty of the patient [41,42,56], and similar concerns could be raised in terms of applicability. Moreover, the case series of Nabeya et al. included a limited sample from a single specific outbreak [57], which could lead to some concerns in terms of ROB, with no issues in terms of applicability.

When dealing with the index test, all sampled tests were potentially affected by some degree of bias. In fact, all sampled patients received their CT scans after the previous laboratory diagnosis of viral LRTI, including the presumptive etiological diagnosis. As a consequence, the readers could hardly be considered “blind” with regard to the underlying disorder [41,42,52,53,54,55,56,57,58,59]. On the contrary, when focusing on the applicability issues, no significant concerns could be raised, as all studies were based on machineries that could be defined as standard by the timeframe of the report.

Regarding the reference standards, eight of 10 studies relied on highly sensitive and specific tests including RT-qPCR, while, in the studies of Escuissato et al. [41] and Jia et al. [53], diagnosis of RSV infection was achieved by means of immunofluorescence. This shortcoming could be reasonably confirmed by the real-world experience, as modern studies would reasonably rely on more accurate testing by means of point-of-care (POC) or real-time quantitative polymerase chain reaction (RT-qPCR) tests. Lastly, when dealing with the theme of patient flow, pre-pandemic studies were reasonably free from time constraints. On the contrary, we cannot rule out that the requirement of the SARS-CoV-2 pandemic may have increased the risk of misclassification of the CT studies [53,54,58].

As shown in Table 3, the formal reporting from the various studies was quite inconstant: features of organizing pneumonia and ground glass opacities were included in the reports from all includes articles, while pleural features were included in nine reports, and the notation for nodular lesions was included in eight articles, with septal thickening and tree-in-bud notation included in seven studies. Interestingly, features compatible with the notation of crazy paving were included in only four studies, while three studies described whether the involvement could be defined as extensive and/or multifocal vs. focal, and two studies described whether it could be defined as unilateral or bilateral. Interestingly, nine articles reported a share of sampled CT studies that appeared normal at the analysis.

Briefly, around 23.4% had normal findings at chest CT scans. When dealing with CT scan features, the large majority of samples (76.1%) had bilateral involvement, while an extensive or multifocal pattern was reported by only 16.7% of specimens. GGOs were identified in 35.9% of samples, while nodular lesions were reported in 42.7% of cases, and 34.1% were characterized as tree-in-bud features, but only 5.0% of samples had any sign of crazy paving. Signs of organizing pneumonia were reported by 39.2% of samples, while 35.3% of them had a sign of interstitial pneumonia reported as septal thickening. Furthermore, 23.9% of samples were complicated by pleural effusions.

Interestingly, substantial differences in the imaging features were identified across the age groups. First of all, normal findings were reported only among adults (30.2%) and individuals characterized by immune deficiency (18.3%; *p* = 0.009). Moreover, the latter group was more frequently characterized by features such as GGOs (55.1% vs. 43.0% in adults, and 16.0% in children, *p* < 0.001), nodular lesions (53.7%, vs. 45.9% in adults and none in children/adolescents, *p* < 0.001), and tree-in-bud (40.9% vs. 26.5% in adults, and 27.8% in children, *p* = 0.002). Similarly, signs of organizing pneumonia, septal thickening, and pleural effusions were more frequently reported among individuals affected by any immune deficiency (64.8%, 43.7%, and 50.7%, respectively) than among adults (27.6%, 38.8%, and 12.1%, respectively) and children (23.3%, 10.0%, and no case, respectively; corresponding *p*-values: <0.001, 0.004, and <0.001).

Pooled prevalence rates are reported in Table 4. Briefly, cases of RSV were characterized by normal findings in 16.2% of cases (95% CI 10.0–84.6), and 76.1% (95% CI 64.8–84.6) had at diagnosis signs of bilateral involvement, while 21.4% (95% CI 10.0–40.2) were affected by extensive or multifocal involvement. The most commonly reported imaging feature was identified in signs of organizing pneumonia (33.7%, 95% CI 22.29–47.3), followed by septal thickening (33.2%, 95% CI 21.3–47.0), GGOs (28.0%, 95% CI 14.7–46.8), findings of tree-in-bud (27.4%, 95% CI 14.0–44.7), nodular lesions (27.2%, 95% CI 11.8–51.1), signs of pleural effusions (7.8%, 95% CI 1.1–39.2), and crazy paving (5.0%, 95% CI 1.6–14.4).

Nearly all estimates were characterized by some degree of heterogeneity, which was substantial for findings of GGOs (I^2^ = 80.7%), signs of organizing pneumonia (I^2^ = 70.8%), tree-in-bud anomalies (I^2^ = 67.3%), normal findings (I^2^ = 64.0%), and pleural effusions (I^2^ = 58.2%), while moderate heterogeneity was associated with pooled estimates for septal thickening (I^2^ = 42.6%) and nodular lesions (I^2^ = 41.1%). Interestingly, no I^2^ statistics were calculated for signs of bilateral involvement, extension of the pulmonary involvement, and signs of crazy paving because of the limited number of retrieved studies (No. = 2, 3, and 4, respectively).

**Figure A2:**
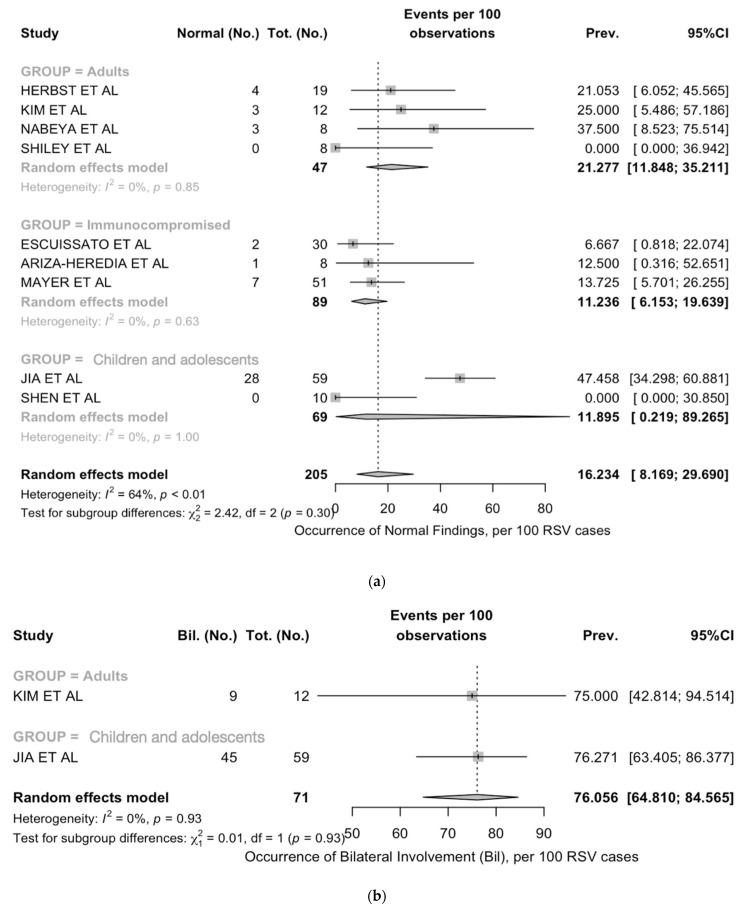

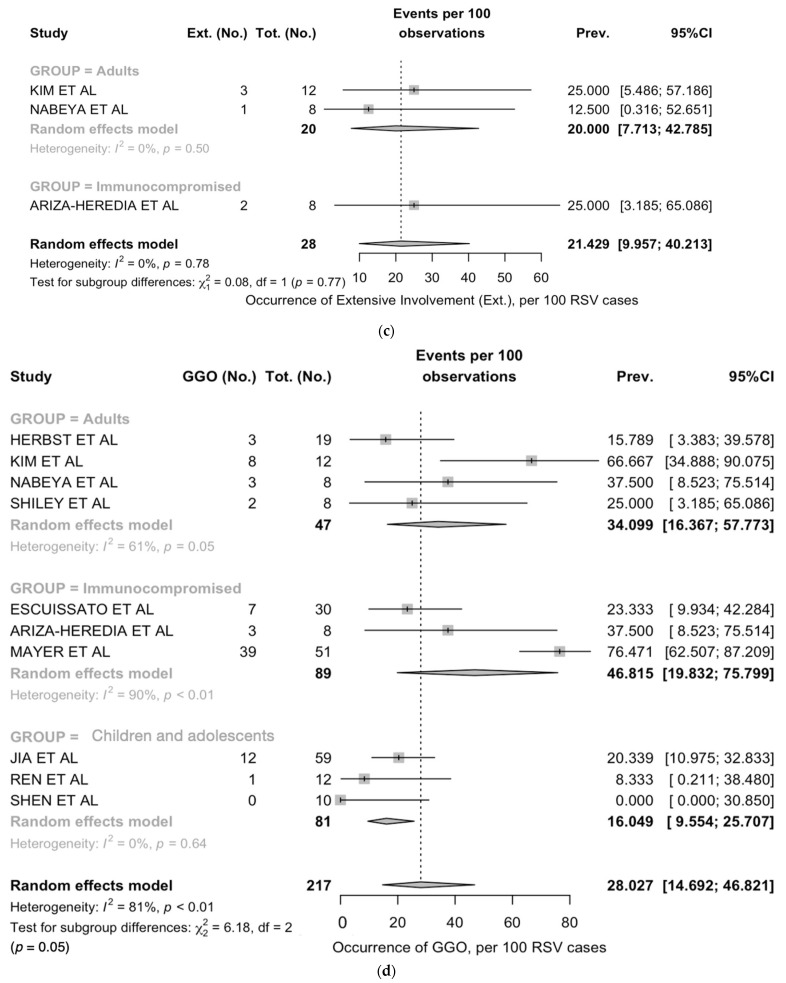

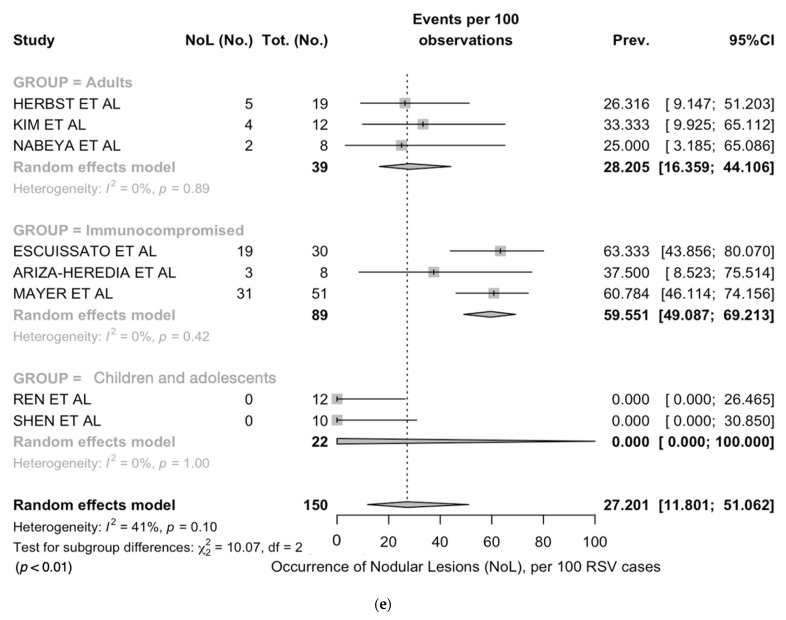

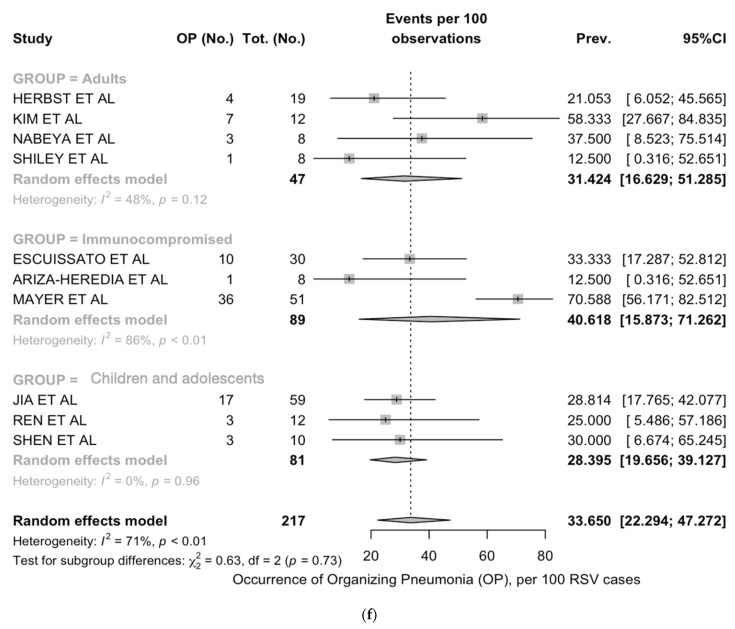

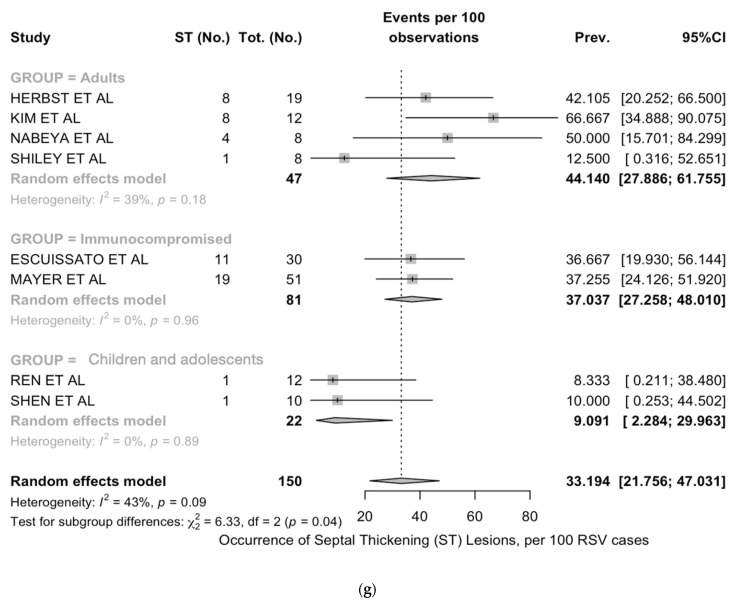

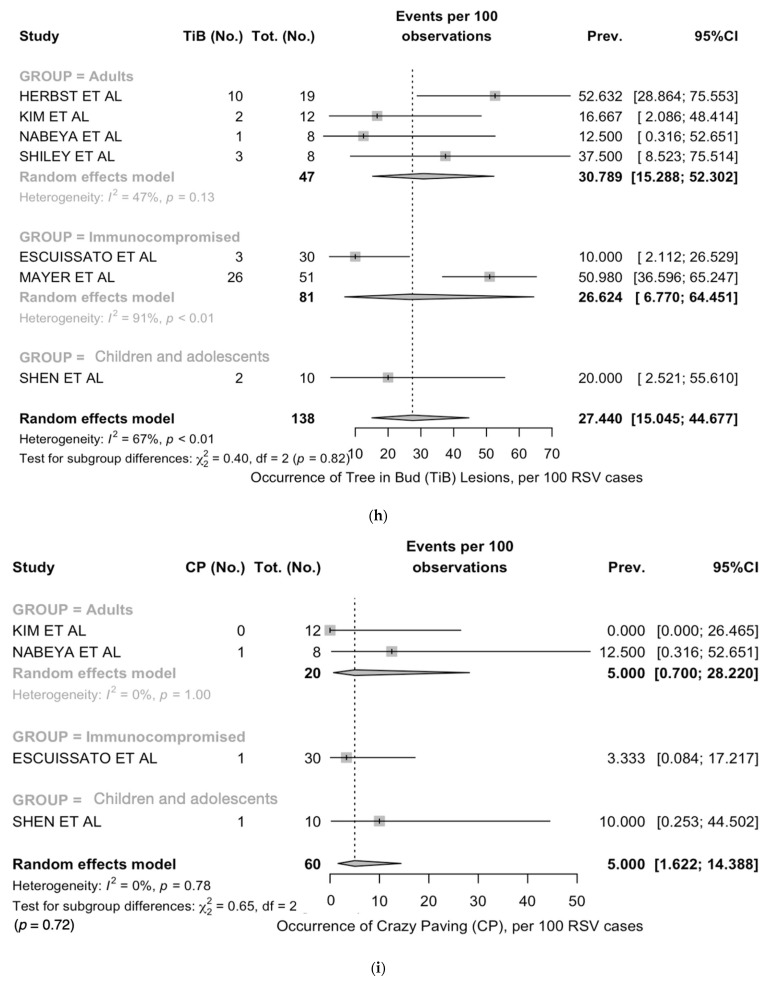

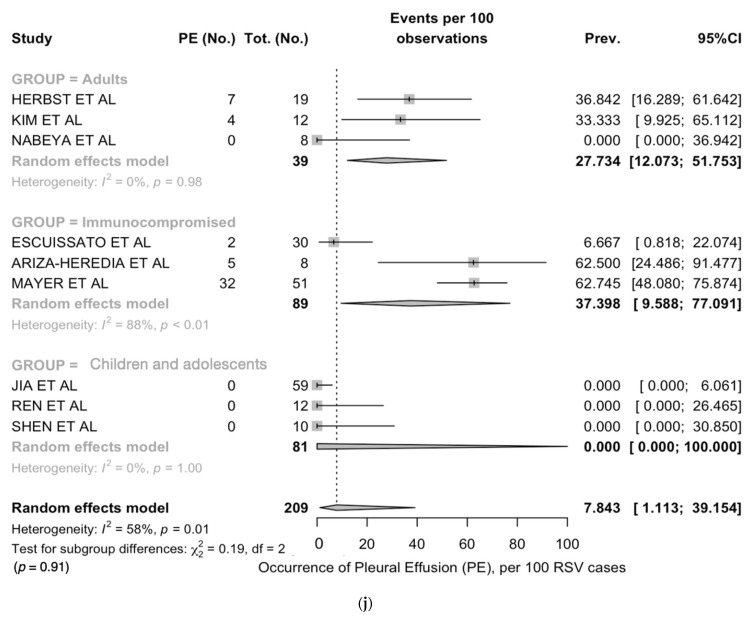
Forest plots of the main findings included in the meta-analysis [41,42,50,52,53,54,55,56,57,58]: (**a**) normal findings; (**b**) bilateral involvement; (**c**) extensive involvement; (**d**) ground glass opacities; (**e**) nodular lesions; (**f**) organizing pneumonia; (**g**) septal thickening; (**h**) tree-in-bud; (**i**) crazy paving; (**j**) pleural effusions.

Subgroup analyses based on the age groups stressed the heterogeneous prevalence rates of the assessed signs and symptoms (Table 5). Corresponding forest plots are reported in full detail in Figure A2.

For instance, pleural effusions were quite rare among children/adolescents (pooled prevalence rate, 0.0%, 95% CI 0.0–100), being reported in around 27.7% (95% CI 12.1–51.8) of RSV cases in adults, peaking among individuals affected by immune deficiencies (38.4%, 95% CI 9.6–77.1). Similarly, nodular lesions, GGOs and signs of organizing pneumonia peaked in cases affected by immune deficiency status (59.5%, 46.8%, and 40.6% respectively), reporting the lowest estimates in children/adolescents (0.0%, 16.0%, and 28.4% respectively), and intermediate figures in otherwise healthy adults (28.2%, 34.1%, and 31.4%). On the contrary, signs of septal thickening and tree-in-bud peaked in adults (44.1% and 30.8%), being followed by individuals affected by immune deficiency status (37.0% and 26.6%) and children/adolescents (9.1%, and 20.0%). In this regard, it should be stressed that the estimates for tree-in-bud features in children/adolescents were calculated on a single study. Regarding bilateral involvement, figures for adults affected by immune deficiency were unavailable, while only one estimate was available for both children/adolescents (76.3%) and adults (75.0%). Similarly, pooled estimates of extensive/multifocal involvement were lacking in reports for children/adolescents, and only one estimate was retrieved for individuals affected by immune deficiency. For crazy paving, estimates in children/adolescents and immune deficiency were based on a single report.

**Figure 3:**
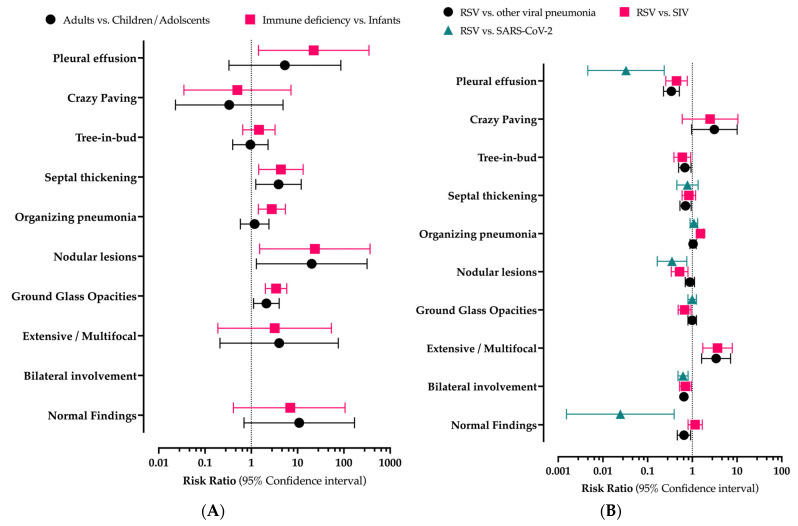
Crude risk ratio (cRR) for main findings of respiratory syncytial infection at chest tomography, as reported by age groups (**A**) and compared to other viral infections (**B**) in the 10 studies included in the systematic review and meta-analysis.

As shown in Figure 3A, when children/adolescents were assumed as the reference group, GGOs were more frequently reported in adults (cRR 2.121, 95% CI 1.121–4.013) and individuals affected by immune deficiency (cRR 2.016, 95% CI 3.430–5.841), as well as nodular lesions (cRR 20.197, 95% CI 1.286–317.082 in adults, and cRR 23.642, 95% CI 1.513–369.496 among individuals affected by immune deficiency). Interestingly, while signs of septal thickening were similarly more frequently reported among adults (cRR 3.878, 95% CI 1.253–12.000) and immune-deficient subjects (cRR 4.366, 95% CI 1.445–13.190) than among children/adolescents, organizing pneumonia was increasingly scored by only individuals affected by immune deficiency (cRR 2.777, 95% CI 1.420–5.461).

As shown in Table 6, when estimates for RSV were compared to the features associated with other viral pathogens, the share of normal scans was similar between RSV and seasonal influenza (23.4% for RSV vs. 27.1% for seasonal influenza, *p* = 0.513), and substantially higher in RSV compared to COVID-19 (no normal scans out of 87 samples, *p* < 0.001), and to all viral infections (15.1%, *p* = 0.019). Assuming RSV cases as the reference group, a corresponding cRR 0.025 (95% CI 0.002–0.394) was reported for SARS-CoV-2 cases, compared to cRR 0.650 (95% CI 0.462–0.916) for all viral pulmonary infections (Figure 3B and Table A2).

Regarding the extent of pulmonary involvement, it was more commonly bilateral in RSV than in diagnoses of COVID-19 (76.1% vs. 47.1%, *p* < 0.001; cRR 0.620, 95% CI 0.479–0.802), seasonal influenza (53.3%, *p* = 0.019; cRR 0.701, 95% CI 0.518–0.949), and all viral infections (49.3%, *p* < 0.001; cRR 0.648, 95% CI 0.535–0.800), while an extensive or multifocal involvement was less frequently reported in RSV compared to seasonal influenza and all other viral infections (21.4% vs. 78.6% and 73.1%; *p* = 0.001 and *p* < 0.001, respectively), whose corresponding crude risk ratio was estimated at 3.667 (95% CI 1.714–7.842) and 3.410 (95% CI 1.616–7.195), respectively.

Interestingly enough, GGOs were more frequently reported among cases of SARS-CoV-2 infection (46.5%) than among those of RSV (35.9%), but the difference was not significant (*p* = 0.051; cRR 0.995, 95% CI 0.801–1.237), as well as for studies on viral infections (35.8%, *p* = 0.967; cRR 0.995, 95% CI 0.801–1.237). Conversely, a substantially lower occurrence was identified in studies for seasonal influenza (24.2%, *p* = 0.019; cRR 0.673, 95% CI 0.483–0.938). Nodular lesions were more frequently reported among RSV cases (42.7%) than among cases of COVID-19 (15.0%, *p* = 0.002; cRR 0.351, 95% CI 0.164–0.752), and seasonal influenza (22.8%, *p* = 0.002; CRR 0.520, 95% CI 0.339–0.799), while no substantial differences were scored for all non-RSV viral infections (37.6%, *p* = 0.358; cRR 0.882, 95% CI 0.695–1.119).

As the features for tree-in-bud were not reported in studies on COVID-19 cases, comparisons were only made between RSV and seasonal influenza (34.1% vs. 20.4, cRR 0.597, 95% CI 0.338–0.921) and all non-RSV viral infections (23.2%, cRR 0.681, 95% CI 0.495–0.938); in both cases, the difference was significant (*p* = 0.023 and *p* = 0.027, respectively).

Signs of organizing pneumonia were less frequently reported among studies for RSV than among those on seasonal influenza (cRR 86.3% vs. 39.2%; 1.522, 95% CI 1.306–1.775), SARS-CoV-2 infections (61.4%; cRR 1.084, 95% CI 0.891–1.319), and all respiratory infections (59.0%; cRR 1.041, 95% CI 0.887–1.222), but the difference was significant for influenza cases only (*p* < 0.001). Conversely, septal thickening was more frequently reported in RSV cases than among cases of COVID-19 (35.3% vs. 27.5%; cRR 0.778, 95% CI 0.450–1.346), seasonal influenza (29.5%; cRR 0.835, 95% CI 0.589–1.184), and all respiratory infections (24.9%; cRR 0.705, 95% CI 0.529–0.941), but the difference was significant only for the latter group (*p* = 0.026). No significant differences were also reported for signs of crazy paving, not only for seasonal influenza (5.0% prevalence in RSV vs. 10.6%, *p* = 0.379; cRR 2.500, 95% CI 0.596–10.490), but also for all non-RSV viral pneumonia (15.6%, *p* = 0.067; cRR 3.111, 95% CI 0.965–10.033).

Pleural effusion was more frequently reported in RSV cases than in cases of SARS-CoV-2 infections (23.9% vs. 0.8%, *p* < 0.001; cRR 0.033, 95% CI 0.005–0.235), seasonal influenza (10.6%, *p* = 0.003; cRR 0.443, 95% CI 0.255–0.769), and for all non-RSV respiratory infections (8.1%, *p* < 0.001; cRR 0.341, 95% CI 0.227–0.511).

Pooled risk ratios were also calculated through a meta-analytical approach and by taking in account the age groups in order to reconcile with the characteristics of the collected samples. Results are reported in Table 7, while their estimates for heterogeneity are reported in Table A3.

Briefly, when the age group was included in the analyses, the main findings associated with RSV were the higher occurrence of bilateral involvement in seasonal influenza (pRR 1.425, 95% CI 1.047–1.940) and in all viral infections (pRR 1.542, 95% CI 1.248–1.904), with the latter being also characterized by a higher risk of signs of septal thickening (pRR 1.433, 95% CI 1.019–2.015), and lower occurrence of GGOs (pRR 0.636, 95% CI 0.481–0.841), nodular lesions (pRR 0.740, 95% CI 0.572–0.959), and signs of organizing pneumonia (pRR 0.713, 95% CI 0.549–0.925). On the contrary, seasonal influenza infections were characterized by a higher occurrence of tree-in-bud opacities compared to RSV cases (pRR 1.870, 95% CI 1.125–3.108).

**Figure A3:**
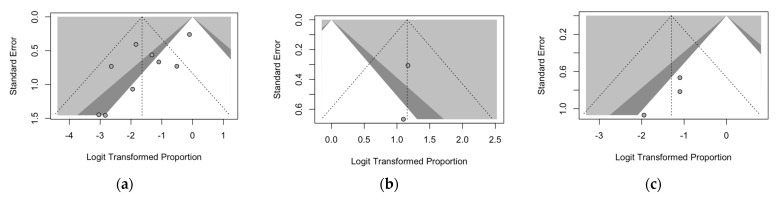

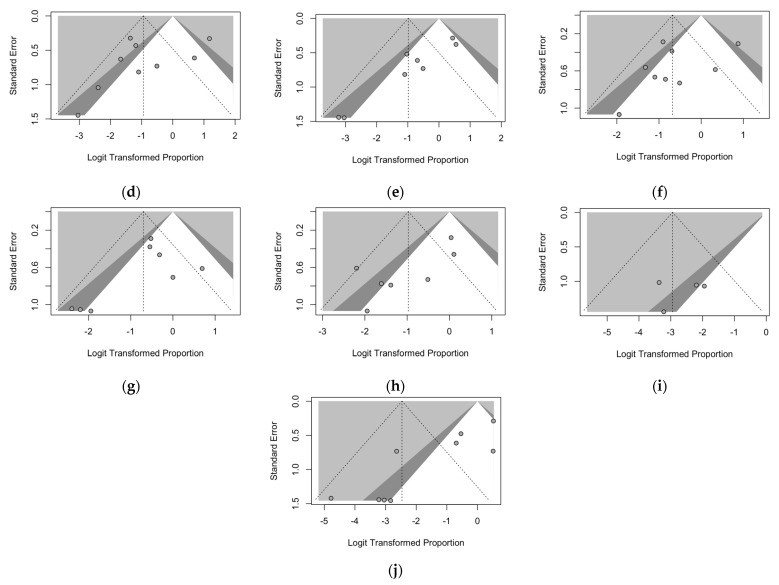
Funnel plots for the findings included in the meta-analysis: (**a**) normal findings; (**b**) bilateral involvement; (**c**) extensive involvement; (**d**) ground glass opacities; (**e**) nodular lesions; (**f**) organizing pneumonia; (**g**) septal thickening; (**h**) tree-in-bud; (**i**) crazy paving; (**j**) pleural effusions.

**Figure A4:**
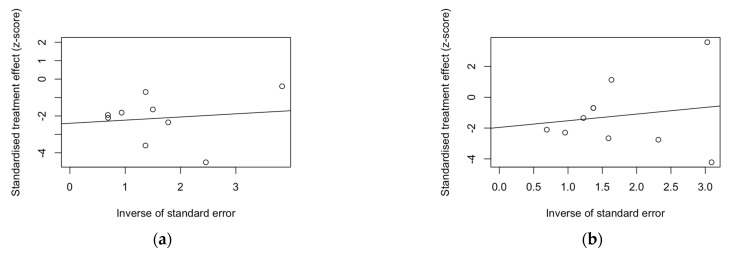

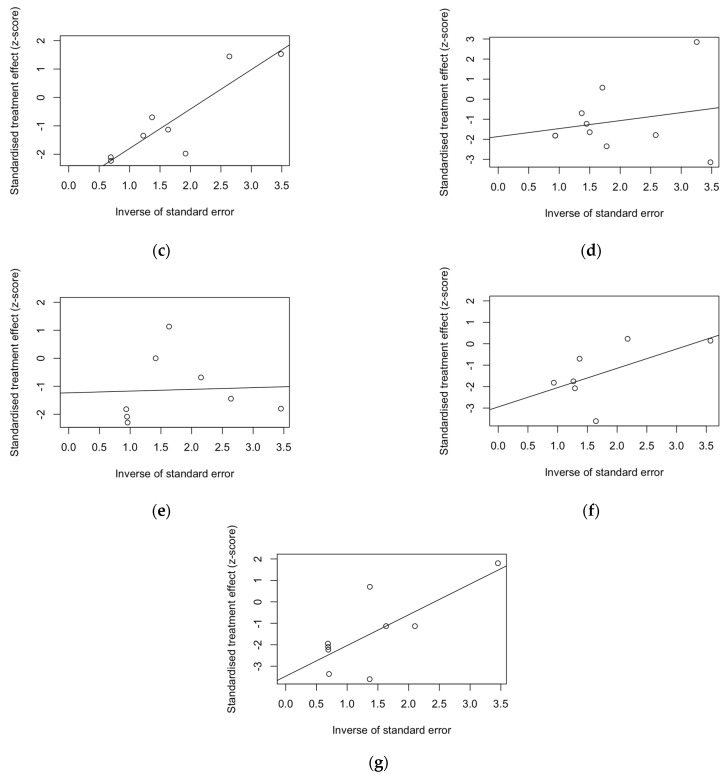
Radial plots for the findings included in the meta-analysis: (**a**) normal findings; (**b**) ground glass opacities; (**c**) nodular lesions; (**d**) organizing pneumonia; (**e**) septal thickening; (**f**) tree-in-bud; (**g**) pleural effusions.

The presence of publication bias was evaluated using funnel plots, while small study bias was assessed through the calculation of radial plots. Funnel plots are reported in Figure A3, while corresponding radial plots are presented as Figure A4, and the detailed results of Egger’s test are summarized in Table A4. In a funnel plot, studies’ effect sizes are plotted against their standard errors; each point represents a separate study, and their asymmetrical distribution at visual inspection is considered suggestive of publication bias (i.e., publication depending not only on the quality of the research, but also on the hypothesis tested, and the significance and direction of detected effects). As suggested by Figure A3, most of the assessed findings were affected by some degree of publication bias, and such subjective evidence from the funnel plot was only confirmed after the regression test. In fact, Egger’s test ruled out publication bias for the findings represented by GGOs (Figure A3d; intercept = 0.434, standard error [SE] = 0.941, t = −1.10; *p* = 0.305), organizing pneumonia (Figure A3f; intercept = 0.399, SE = 0.652, t = −1.37; *p* = 0.207), and septal thickening (Figure A3g; intercept = 0.062, SE = 0.527, t = −1.19; *p* = 0.279). On the other hand, in radial plots (Figure A4), corresponding estimates for GGOs, organizing pneumonia, and septal thickening (Figure A4d,f,g, respectively) were not substantially scattered across the regression line, suggesting a potential small study effect for these very same findings.

## 4. Discussion

In this systematic review and meta-analysis on a total of 10 studies and 1276 index cases collected between 1993 and 2020, the most prevalent CT scan finding for RSV was the bilateral involvement of the lungs (76.1%, 95% CI 64.8–84.6), with a predominant pattern of organizing pneumonia (33.7%, 95% CI 22.3–47.3), followed by signs of septal thickening (33.2%, 95% CI 21.8–47.0), GGOs (28.0%, 95% CI 14.7–46.8), nodular lesions (27.2%, 95% CI 11.8–51.1), and tree-in-bud (27.4%, 95% CI 15.0–44.7). Sings of crazy paving (i.e., ground glass opacities with superimposed interlobular septal thickening and intralobular septal thickening), were rarer but still notably reported (5.0%, 95% CI 1.6–14.4). Interestingly enough, normal findings were identified in 16.2% (95% CI 8.2–29.7) of all cases, suggesting that a considerable share of sampled individuals may fail to be properly diagnosed even on CT scans. Collectively, such features are highly consistent with some previous reports [27,50], as well as the relatively low rates for pleural effusion (7.8%, 95% CI 1.1–39.2).

Moreover, collected features were highly heterogeneous by sampling strategy, sample size, and assessed age groups, as suggested by the detailed assessment of the risk of bias and heterogeneity across all the included studies. Therefore, when addressing our results, a precautionary approach is forcibly required. Substantial differences were reported across the various age groups, and our study suggests that RSV infections may develop quite different imaging features in healthy children, healthy adults, and immunodeficient individuals [42,56]. In fact, signs of nodular lesions, GGOs, and tree-in-bud opacities, as well as signs of septal thickening, were more frequently reported in healthy adults than in children/adolescents [27,39,40,41,60,61,62], while individuals affected by immunodeficiency were also characterized by signs of organizing pneumonia and pleural effusions, otherwise rare in children and healthy adults [41,42,51,63]. These age-specific features of RSV infections should be carefully taken in account for achieving an appropriate and timely differential diagnosis with other respiratory pathogens [27]. Compared to other viral respiratory infections, RSV imaging was also characterized by a lower occurrence of bilateral involvement and signs of septal thickening, with higher occurrence of GGOs, nodular lesions, and signs of organizing pneumonia. In other words, our results could seemingly suggest that RSV infections are more frequently bilateral but less extensive than other viral respiratory pathogens. Interestingly, seasonal influenza infections were associated with a higher occurrence of tree-in-bud opacities compared to RSV cases (pRR 1.870, 95% CI 1.125 to 3.108).

In other words, our results are consistent with previous reports stressing the large overlap in imaging features of chest CT findings for viral pneumonia [27,36], particularly severe influenza [64] and SARS-CoV-2 infections [65], mostly including GGOs and signs of consolidations [52,53,54,55,59]. Moreover, the noticeable share of normal scans hints that the sensitivity of CT scans for RSV could even be worse than that associated with SARS-CoV-2 during the early stage of the COVID-19 pandemic, when 18% of milder cases and no more than 3% of severe cases usually exhibited normal features [66,67], somehow comparable to the performances reported in pediatric cases of COVID-19 [46].

The precautionary approach we forcibly stress when dealing with our results is particularly significant when taking into account the age groups in the meta-analysis approach. In fact, a quite different pattern was identified. First of all, the bilateral involvement was less frequently reported in RSV cases than among other non-RSV viral infections, but these results were reasonably affected by the reduced sample size; in fact, only two studies assessed this specific feature, and both reports did not include individuals affected by immune deficiencies [53,55]. Second, diagnosis of RSV was characterized by findings such as GGOs, nodular lesions, and signs of organizing pneumonia. GGOs are a common feature of several viral infections, resulting from a previous invasion and viral replication in the alveolar epithelium, which in turn result in the alveolar cavity leaking under the pleural or around the peribronchovascular regions [45,46,65]. These findings were only partially expected; even though the high rate of GGOs, particularly among immunocompromised individuals, has been previously described [27,49,50,52,59,68], we were unable to report high rates of septal thickening and bronchial wall impairment, signs that were previously described as quite common in RSV cases compared to other viral pathogens [27,46,69]. Seasonal influenza was characterized by a higher occurrence of tree-in-bud opacities than in RSV cases, i.e., the opposite to that suggested by a more general approach to the collected data.

Several explanations could be provided. For one, a substantial selection bias reasonably affected our estimates, as suggested by the correlation analysis, which suggested a higher share of RSV cases among smaller samples. As RSV is a very common infection, particularly among infants aged 2 years or less [24,26,70,71,72,73], the large majority of incident cases were reasonably managed as outpatient cases, not requiring any imaging and, therefore, not being included in the present estimates. Moreover, even among hospitalized (i.e., more severe) cases, CT scans were performed only in highly selected (and not necessarily representative cases) cases, in order to better characterize severe respiratory impairment and/or rule out other respiratory infections that would require a specific follow-up, potentially including antimicrobic therapy, otherwise useless in RSV cases [39,40,49,59,74]. In fact, 17.0% of the 1224 CT scan studies included in the pooled sampled population were taken in RSV cases, which in turn represented only a small fraction of all individuals followed by parent healthcare providers. Sampling issues were particularly significant when dealing with studies reporting on pediatric-age cases that were mostly performed in mainland China, during the first stages of the SARS-CoV-2 pandemic, when chest CT scans were the only available option for achieving a reliable and timely diagnosis of COVID-19 [53,54,58]. In the study of Jia et al. [53], a total of 59 RSV cases were reported from 164 CT studies that in turn were retrieved from a way larger population of 947 pediatric pneumonia patients (i.e., 6.2% of the original study population). The report from Ren et al. [54] encompassed a total of 12 RSV cases, from 384 CT studies, but the original study population included 4335 children with community-acquired pneumonia (i.e., <0.3% of the original population) [53,54,58]. In other words, it is reasonable that sampled cases may eventually reflect a highly selected group of infant patients, with a certain oversampling of cases characterized by more extensive and severe LRTI, whose representativity of the general pediatric population may, therefore, be questioned.

Similarly, it should be stressed that, even though RSV infections in adults and elderly are quite commoner than usually acknowledged [75,76,77,78,79,80,81], as is the diagnostic referral to chest CT scans, the substantial oversampling of more complicated cases again cannot be ruled out, particularly in light of the inclusion criteria of retrieved studies. As CT studies were usually performed on patients with a previous diagnosis of LRTI [52,55,57,59], most milder cases were not included in the final sample. For example, a total of 263 RSV cases were initially included in the report from Ariza-Heredia et al. [42], but only 24 (i.e., 9.1%) received diagnostic imaging. Similarly, the study of Escuissato et al. [41] collected from a parent population of 774 subjects who had undergone bone marrow transplantation, but only included a total of 111 cases of pulmonary infections (14.3%), 33 of them being RSV cases (4.3% of the original population), while the study from Mayer et al. did not provide a detailed estimate of the parent population [56].

Moreover, the pathological pattern associated with LRTI, including RSV infection, not only evolves over time but is also substantially affected by the different stages of organ maturation in the various life phases [82,83]. RSV-associated bronchiolitis (i.e., distal bronchiole inflammation and obstruction) reduces the airflow into the small airways, leading to an impairment of the exhalation capacity, which in turn leads to lung hyper-expansion, alteration in the lung function, and increased production of mucus, with increasing occurrence of atelectasis [82,84,85]. Subsequent infection of the alveolar epithelium then leads to pneumonia, which in turn may trigger distal airway inflammation that leads to the increasing impairment of the gas exchanges [82,85]. Corresponding imaging features depend on the time course of the infection when the patient is scanned and on the maturation stage of the airways [27]. As a consequence, an appropriate comparison of CT scan features would require the recruitment of patients in properly balanced age groups at a similar stage of RSV infection, and retrieved studies were in fact quite heterogeneous. For instance, while scans from the study of Herbst et al. [52] were performed within the first 7 days from the positive diagnosis for the viral pathogens, this timeframe increased to 14 days in the report from Kim et al. [55], and no detailed information was provided by remaining reports [50,57,59].

Studies performed in individuals affected by immunodeficiency were affected by similar shortcomings [41,42,56], but the very same background characteristics of sampled patients was also quite heterogeneous. In fact, this subgroup encompassed patients affected by iatrogenic immunodeficiency following bone marrow [41] or solid organ transplantation [42], as well as immunodeficiency associated with malignancies and autoimmune diseases [56]. Nonetheless, it should be stressed that immunocompromised individuals are at high risk for developing respiratory infections from a vast array of different pathogens. As a consequence, not only are immunocompromised individuals with signs of any respiratory disorder more likely to be studied through CT scans [41,56], but the reported features may also result from any possible overlap with other synchronous and/or diachronous infections [27,56].

*Strengths and Limitations*. Despite the potential significance as a guide for professionals involved in the differential diagnosis of LRTI, our study was affected by substantial shortcomings. First and foremost, the reliability of the present meta-analysis was substantially affected by the heterogeneity of the included studies (i.e., the variation in study outcomes between studies). According to current guidelines [43,86,87], a precautionary approach should be applied when dealing with I^2^ statistics greater than 50%, as for the analyses we performed on signs of GGO (81%), organizing pneumonia (71%), tree-in-bud (67%), and pleural effusion (58%). In order to address this potential shortcoming, we performed a sub-analysis of collected data by patient groups; even though this approach led to a better characterization of specific patterns, it also stressed how certain features were only reported by a very restricted subset of studies, thus being of limited generalizability.

Second, the number of studies we were able to retrieve was relatively limited, particularly when compared to the gargantuan size of the research output on COVID-19 and influenza, which is reasonably explainable through the quite limited role of CT scans in the diagnosis of viral pneumonia before the inception of the SARS-CoV-2 pandemic [36,45,46]. Nonetheless, even the studies performed during the SARS-CoV-2 pandemic require a critical appraisal; because of extensive use of imaging examinations during the early stages of the pandemic, eventual figures could have been radically inflated, with a substantial overestimation of the actual figures of RSV pulmonary complications in children and adolescents. In other words, the overall quality of sampled studies and the population they targeted were strikingly heterogeneous, as stressed not only by qualitative assessment, but also by quantitative analyses, eventually recommending a quite careful generalization of our results.

Third, the majority of studies on adults and immunocompromised individuals were performed before the inception of the SARS-CoV-2 pandemic [41,42,52,55,56,57,59]; hence, differential diagnosis with COVID-19 was not properly provided, impairing the reliability of our estimates on diagnostic accuracy for reported imaging features. On the contrary, all of the retrieved pediatric studies were performed during the early stages of the pandemic, from mainland China only [53,54,58]. As these studies obviously anticipated the ongoing post-SARS-CoV-2 pandemic RSV resurgence [88,89,90,91,92,93,94,95], the post hoc diagnostic performances of assessed CT scans are limitedly comparable with the current international situation.

Fourth, when dealing with the comparisons with other respiratory pathogens, particularly with SARS-CoV-2, all the reported studies were performed before the global delivery of SARS-CoV-2 vaccines, as well as before the emergence of the Omicron variant of concern of SARS-CoV-2. Compared to the Wuhan strain, Omicron and its subvariants have been allegedly associated with milder clinical features, usually explained through a quite different pattern of viral invasion that ultimately leads to a reduced rate of LRTI [96,97,98]. As SARS-CoV-2 vaccines, particularly mRNA-based vaccines, have retained their substantial efficacy in avoiding severe complications [99,100], even breakthrough infections appear to be less invasive to the deeper airways than in earlier stages of the pandemic. As a consequence, the share of abnormal scans in subjects affected by SARS-CoV-2 infection has radically decreased over time, particularly in children [45,46].

Fifth, current guidelines on the management of RSV prioritize diagnostic options such as ultrasonographic studies over radiographic procedures [37,101,102,103,104,105]. Even though CT scans are minimally invasive and the procedure can be conducted quickly, their use for the diagnosis of RSV cases would lead to inevitable radiation exposure of subjects that are particularly sensitive to radiations including children and immunocompromised individuals; according to the estimates of the National Health Institute, despite CT scans comprising up to about 12% of diagnostic radiological procedures in the USA, it is estimated that they account for approximately 49% of the US population’s collective dose from all medical X-ray examinations [106,107,108]. As a consequence, the use of CT scans for the study of RSV has usually been restricted to only a few severe cases where there is a need to assess complications, particularly secondary bacterial infections. Not coincidentally, the large majority of retrieved studies on children/adolescents were performed during the SARS-CoV-2 pandemic; even in adults, a substantial share of studies was associated with background conditions such as bone marrow and solid organ transplantations. Therefore, this fact alone could have introduced a significant selection bias for all studies investigating tomography in RSV infections.

All the retrieved studies were performed in settings where the only available preventative option for RSV was represented by palivizumab, a humanized monoclonal antibody (SYNAGIS^®^; USA approval 1998, EU approval 1999) [9,109,110,111,112,113]. Palivizumab is delivered through monthly injections of a weight-dependent dose (i.e., 15 mg/kg) during the months characterized by high circulation of pathogen (“RSV season”), with up to five consecutive doses [9,10,110,111,112,114]. Despite its proven efficacy, this is a relatively expensive medication, as the cost for a 100 mg vial usually ranges from 904 to 1866 USD [4,5,24,25,26,28], and current guidelines have restricted its delivery to high-risk groups [8,14,113,115,116,117], including (a) infants born at ≤35 weeks of gestational age (wGA), (b) children < 2 years of age affected by chronic lung disease of prematurity (CLD), and (c) children < 2 years of age affected by hemodynamically significant congenital heart disease (CHD) [118,119,120]. As a consequence, even though the previous delivery of palivizumab was not reported across the retrieved studies, its actual role may be acknowledged as quite limited. Nevertheless, an extended half-life recombinant mAb, i.e., nirsevimab (MEDI8897; commercial name: Beyfortus^®^), has recently been approved in the EU for the prevention of RSV-associated LRTI in newborns and infants from birth during their first RSV season [121,122,123]. Because of its extended half-life, the recommended dose of nirsevimab is a single intramuscular injection of 50 mg for infants with body weight < 5 kg, and a single intramuscular injection of 100 mg for infants with body weight ≥ 5 kg [10,26,124,125], being potentially more affordable in terms of direct and indirect costs, which in turn could increase the share of targeted individuals in the next few years. As RSV vaccines are similarly under an advanced stage of development [2,14,26,76,126], and as adult formulates have recently been licensed by FDA, our report not only potentially provides guidance on the imaging features of incident RSV cases [21,22,127], but could represent a real-world picture taken before the extensive implementation of preventive interventions potentially able to radically change the natural history of viral pneumonia associated with RSV infections.

## 5. Conclusions

In conclusion, RSV viral pneumonia was characterized by nonspecific findings such as GGOs, signs of consolidation and septal thickening, and nodular lesions. We noticed some substantial heterogeneities in their distribution across age groups, but the potential selection bias urges for a precautionary appraisal of such features. Despite the reported higher occurrence of some findings, particularly GGOs, RSV and non-RSV viral pneumonia have mostly overlapped features on CT scans. As a consequence, medical professionals should exercise substantial caution when referring to this imaging as a diagnostic option for RSV-associated viral pneumonia.

## Figures and Tables

**Table 1:** PICO worksheet modified for studies on diagnostic tests [44].

Item	Definition
Population of interest	Children and adults with diagnosis of respiratory infection
Investigated test result	Report of the following imaging features: bronchitis, alveolar involvement, tree-in-bud, bronchial wall thickening, ground glass opacities, nodules, consolidation, pleural effusions
Comparator test result	Imaging of features of respiratory syncytial virus (RSV) infections compared to other viral respiratory infections
Outcome	Association of imaging features with RSV infection

**Table 2:** Summary of studies included in the quantitative analyses.

Authors	Year	Timeframe	Country	Patients	Category	Diagnosis of RSV	Total Sample (No.)	RSV (No., %)	No Signs (No., %)
Ariza-Heredia et al. [42]	2012	1 Januaty 1997 31 December 2009	USA	All adult patients who underwent solid organ transplant and were positive for RSV at either antigen or RT-qPCR	Immunocompromised	RT-qPCR/antigen testing	24	8, 33.3%	1, 12.5%
Escuissato et al. [41]	2005	January 1993 December 2003	Brazil	All patients (No. 111) with a proven pulmonary infection after bone marrow transplatation and HR-CT within 24 h from the admission	Immunocompromised	Immunofluorescence	111	30, 27.0%	6, 20.0%
Herbst et al. [52]	2013	15 November 2005 15 July 2011	USA	All patients from 2 university-based hospitals having received testing for respiratory pathogens and admitted for LRTI and undergone CT within 7 days from the positive diagnosis	Adults (age ≥ 18 years)	RT-qPCR	113	19, 16.8%	4, 21.1%
Jia et al. [53]	2021	16 January 2020 25 February 2020	China, mainland	All pediatric patients (No. 947) having been assessed in 30 hospitals from 4 provinces for pneumonia	Children/adolescents (age < 18 years)	Immunofluorescence	164	59, 36.0%	28, 47.5%
Kim et al. [55]	2016	January 1998 December 2014	Republic of Korea	All adult patients who underwent BAL and were diagnosed with LRTI caused by PIV, influenza virus or RSV, within 14 days from diagnosis	Adults (age ≥ 16 years)	RT-qPCR/immunofluorescence	139	12, 8.6%	3, 25.0%
Mayer et al. [56]	2014	November 2011 July 2012	Germany	All immunocompromized patients from the same clinic having received a CT scan within 24 h after the beginning of clinical symptoms	Immunocompromised	RT-qPCR/antigen testing/immunofluorescence	132	51, 38.6%	7, 13.7%
Nabeya et al. [57]	2020	2014	Japan	Eight patients who, during the outbreak of 2014, received CT scans and were genetically related in terms of pathogens	Adults (age ≥ 18 years)	RT-qPCR	8	8, 100%	3, 37.5%
Ren et al. [54]	2021	15 December 2019 15 March 2020	China, mainland	All consecutive children diagnosed with community-acquired pneumonia	Children/adolescents (age < 18 years)	RT-qPCR	384	12, 3.2%	n.a.
Shen et al. [58]	2020	23 January 2020 20 March 2020	China, mainland	All consecutive children diagnosed with community-acquired pneumonia	Children/adolescents (age < 18 years)	RT-qPCR	159	10, 6.3%	0, 0.0%
Shiley et al. [50]	2010	1 November 2005 31 July 2007	USA	All adults patients with a viral diagnosis, PCR analysis and CT scans	Adults (age ≥ 18 years)	RT-qPCR	42	8, 19.0%	0, 0.0%

**Table 3:** Cumulative occurrence of main findings of respiratory syncytial infection at chest tomography, reported by age groups in the 10 studies included in the systematic review and meta-analysis.

Item	No. of Studies (No./10)	Total	Children/Adolescents (Age < 18 Years)	Adults (Age ≥ 18 Years)	Immune Deficiency	Chi^2^ *p*-Value
Normal findings	9	48/205, 23.4%	0/18, 0.0%	35/116, 30.2%	13/71, 18.3%	0.009
Bilateral involvement	2	54/71, 76.1%	n.a.	54/71, 76.1%	n.a.	n.a.
Extensive/multifocal	3	3/36, 16.7%	0/8, 0.0%	2/8, 25.0%	4/20, 20.0%	0.340
Ground glass opacities	10	78/217, 35.9%	13/81, 16.0%	16/47, 34.0%	49/89, 55.1%	<0.001
Nodular lesions	8	64/150, 42.7%	0/22, 0.0%	28/61, 45.9%	36/67, 53.7%	<0.001
Tree-in-bud	7	47/138, 34.1%	5/18, 27.8%	13/49, 26.5%	29/49, 40.9%	0.002
Organizing pneumonia	10	85/217, 39.2%	7/30, 23.3%	32/116, 27.6%	46/71, 64.8%	<0.001
Septal thickening	7	53/150, 35.3%	3/30, 10.0%	19/49, 38.8%	31/71, 43.7%	0.004
Crazy paving	4	3/60, 5.0%	1/10, 10.0%	1/30, 3.3%	1/20, 5.0%	0.704
Pleural effusion	9	50/209, 23.9%	0/22, 0.0%	14/116, 12.1%	36/71, 50.7%	<0.001

**Table 4:** Pooled prevalence for main findings of respiratory syncytial infection at chest tomography, reported in the 10 studies included in the systematic review and meta-analysis.

Item	Prevalence (No. per 100 Studies)	I^2^	Q	tau^2^	*p*-Value
Normal findings	16.2 (8.2; 29.7)	64.0	22.20	0.745	0.005
Bilateral involvement	76.1 (64.8; 84.6)	0.0	0.01	0.000	0.924
Extensive/multifocal	21.4 (10.0; 40.2)	0.0	0.51	0.000	0.775
Ground glass opacities	28.0 (14.7; 46.8)	80.7	46.66	1.241	<0.001
Nodular lesions	27.2 (11.8, 51.1)	41.1	11.88	1.473	0.1805
Tree-in-bud	27.4 (15.0; 44.7)	67.3	18.34	0.606	0.005
Organizing pneumonia	33.7 (22.3; 47.3)	70.8	30.85	0.481	<0.001
Septal thickening	33.2 (21.8; 47.0)	42.6	12.19	0.303	0.094
Crazy paving	5.0 (1.6; 14.4)	0.0	1.08	0.000	0.783
Pleural effusion	7.8 (1.1; 39.2)	58.2	19.12	6.063	0.014

**Table 5:** Pooled prevalence for main findings of respiratory syncytial infection at chest tomography, reported by age groups in the 10 studies included in the systematic review and meta-analysis.

	Children/Adolescents (Age < 18 Years)		Adults (Age ≥ 18 Years)		Immune Deficiency	
	Prevalence (No. per 100 Studies)	I^2^	Prevalence (No. per 100 Studies)	I^2^	Prevalence (No. per 100 Studies)	I^2^
Normal findings	11.9 (0.2; 89.3)	0%	21.3 (11.8; 35.2)	85%	11.2 (6.2; 19.6)	0%
Bilateral involvement	76.3 (63.4; 86.4)	-	75.0 (42.8; 94.5)	-		
Extensive/multifocal	-	-	20.0 (7.7; 42.8)	0%	25.0 (10.0; 40.2)	-
Ground glass opacities	16.0 (10.0; 25.7)	0%	34.1 (16.4; 57.8)	61%	46.8 (19.8; 75.8)	90%
Nodular lesions	0.0 (0.0; 100)	0%	28.2 (16.4; 44.1)	0%	59.5 (49.1; 69.2)	0%
Tree-in-bud	20.0 (2.5; 55.6)	-	30.8 (15.3; 52.3)	47%	26.6 (6.8; 64.5)	91%
Organizing pneumonia	28.4 (19.7; 39.1)	0%	31.4 (16.6; 51.3)	48%	40.6 (15.9; 71.3)	86%
Septal thickening	9.1 (2.3; 30.0)	0%	44.1 (27.9; 61.8)	39%	37.0 (27.3; 48.0)	0%
Crazy paving	10.0 (0.3; 44.5)	-	5.0 (0.7; 28.2)	0%	3.3 (0.1; 17.2)	-
Pleural effusion	0.0 (0.0; 100)	0%	27.7 (12.1; 51.8)	0%	38.4 (9.6; 77.1)	88%

**Table 6:** Cumulative occurrence of main findings of respiratory syncytial (RSV) infection at chest tomography, compared to the features for COVID-19, seasonal influenza, and all non-RSV viral infections with their corresponding chi-squared test *p*-values, as reported in the 10 studies included in the systematic review and meta-analysis.

Item	RSV (10 Studies)	COVID-19 (3 Studies)	Chi^2^ *p*-Value	Seasonal Influenza (7 Studies)	Chi^2^ *p*-Value	All Non-RSV Viral Infections (7 Studies)	Chi^2^ *p*-Value
Normal findings	48/205, 23.4%	0/87, 0.0%	<0.001	39/144, 27.1%	0.513	58/381, 15.1%	0.019
Bilateral involvement	54/71, 76.1%	41/87, 47.1%	<0.001	24/45, 53.3%	0.019	71/144, 49.3%	<0.001
Extensive/multifocal	6/28, 21.4%	n.a.	n.a.	11/14, 78.6%	0.001	19/26, 73.1%	<0.001
Ground glass opacities	78/217, 35.9%	59/127, 46.5%	0.051	37/153, 24.2%	0.019	157/439, 35.8%	0.967
Nodular lesions	64/150, 42.7%	6/40, 15.0%	0.002	23/101, 22.8%	0.002	108/287, 37.6%	0.358
Tree in bud	47/138, 34.1%	n.a.	n.a.	23/113, 20.4%	0.023	61/263, 23.2%	0.027
Organizing pneumonia	85/150, 39.2%	78/127, 61.4%	0.498	132/153, 86.3%	<0.001	259/439, 59.0%	0.686
Septal thickening	53/150, 35.3%	11/40, 27.5%	0.457	36/122, 29.5%	0.374	80/321, 24.9%	0.026
Crazy paving	3/60, 5.0%	n.a.	n.a.	4/32, 12.5%	0.379	21/135, 15.6%	0.067
Pleural effusion	50/209, 23.9%	1/127, 0.8%	<0.001	14/132, 10.6%	0.003	33/405, 8.1%	<0.001

**Table 7:** Pooled risk ratio for main findings of respiratory syncytial (RSV) infection at chest tomography, compared to the features for COVID-19, seasonal influenza, and all non-RSV viral infections, as reported in the 10 studies included in the systematic review and meta-analysis.

Item	COVID-19	Seasonal Influenza	All Non-RSV Viral Infections
	Pooled Risk Ratio (95% Confidence Interval)		
Normal findings	-	0.785 (0.457; 1.347)	1.737 (0.737; 4.095)
Bilateral involvement	-	1.425 (1.047; 1.940)	1.542 (1.248; 1.904)
Ground glass opacities	0.391 (0.166; 0.922)	0.730 (0.532; 1.003)	0.636 (0.481; 0.841)
Nodular lesions	-	0.937 (0.473; 1.859)	0.740 (0.572; 0.959)
Tree-in-bud	-	1.870 (1.125; 3.108)	1.221 (0.601; 2.479)
Organizing pneumonia	0.964 (0.332; 2.798)	1.447 (0.907; 2.311)	0.713 (0.549; 0.925)
Septal thickening	-	1.024 (0.696; 1.508)	1.433 (1.019; 2.015)
Crazy paving	-	0.778 (0.021; 29.544)	0.341 (0.096; 1.210)
Pleural effusion	1.080 (0.047; 24.881)	1.603 (0.625; 4.110)	1.362 (0.717; 2.587)

## Data Availability

Source data are available upon request to the corresponding author.

## Supplementary Materials

The following supporting information can be downloaded at https://www.mdpi.com/article/10.3390/children10071169/s1, PRISMA 2020 Checklist–Article: Respiratory Syncytial Virus: A systematic review and meta-analysis of tomographic findings (2000–2022). Reference [128] is cited in the supplementary materials.

## Appendix A

**Table A1:** Tabular representation for the QUADAS-2 results.

Study	Risk of Bias				Applicability Concerns		
	Patient Selection	Index Test	Reference Standard	Flow and Timing	Patient Selection	Index Test	Reference Standard
Ariza-Heredia et al. [42]	☹	?	☺	☺	☹	☺	☺
Escuissato et al. [41]	☹	?	☹	☺	☹	☺	☹
Herbst et al. [52]	☺	?	☺	☺	☺	☺	☺
Jia et al. [53]	☺	?	☹	☹	☺	☺	☹
Kim et al. [55]	☺	?	☺	☺	☺	☺	☺
Mayer et al. [56]	☹	?	☺	☺	☹	☺	☺
Nabeya et al. [57]	☹	?	☺	☺	☺	☺	☺
Ren et al. [54]	☺	?	☺	☹	☺	☺	☺
Shen et al. [58]	☺	?	☺	☹	☺	☺	☺
Shiley et al. [50]	☺	?	☺	☺	☺	☺	☺

☺ = low risk; ? = unclear risk; ☹ = high risk.

**Table A2:** Risk ratio for main findings of respiratory syncytial (RSV) infection at chest tomography, compared to the features for COVID-19, seasonal influenza, and all non-RSV viral infections, as reported in the 10 studies included in the systematic review and meta-analysis.

Item	COVID-19 (3 Studies)	Seasonal Influenza (7 Studies)	All Non-RSV Viral Infections (7 Studies)
	Risk Ratio (95% Confidence Interval)		
Normal findings	0.025 (0.002; 0.394)	1.157 (0.803; 1.666)	0.650 (0.462; 0.916)
Bilateral involvement	0.620 (0.479; 0.802)	0.701 (0.518; 0.949)	0.648 (0.535; 0.800)
Extensive/multifocal	n.a.	3.667 (1.714; 7.842)	3.410 (1.616; 7.195)
Ground glass opacities	0.995 (0.801; 1.237)	0.673 (0.483; 0.938)	0.995 (0.801; 1.237)
Nodular lesions	0.351 (0.164; 0.752)	0.520 (0.339; 0.799)	0.882 (0.695; 1.119)
Tree-in-bud	n.a.	0.597 (0.388; 0.921)	0.681 (0.495; 0.938)
Organizing pneumonia	1.084 (0.891; 1.319)	1.522 (1.306; 1.775)	1.041 (0.887; 1.222)
Septal thickening	0.778 (0.450; 1.346)	0.835 (0.589; 1.184)	0.705 (0.529; 0.941)
Crazy paving	n.a.	2.500 (0.596; 10.490)	3.111 (0.965; 10.033)
Pleural effusion	0.033 (0.005; 0.235)	0.443 (0.255; 0.769)	0.341 (0.227; 0.511)

**Table A3:** Heterogeneity assessment for pooled analyses of CT scans features in respiratory syncytial virus compared to COVID-19, seasonal influenza, and all non-RSV viral infections, as reported in the 10 studies included in the systematic review and meta-analysis.

Item	COVID-19				Seasonal Influenza				All Non-RSV Viral Infections			
	I^2^	Q	*p*	t^2^	I^2^	Q	*p*	t^2^	I^2^	Q	*p*	t^2^
Normal findings	-	-	-	-	42.8	5.25	0.155	0.066	58.4	9.63	0.047	0.460
Bilateral involvement	-	-	-	-	0.0	0.12	0.729	0.001	0.0	0.21	0.647	0.001
Ground glass opacities	22.9	1.30	0.254	0.389	0.0	1.45	0.835	0.001	0.0	3.46	0.749	0.001
Nodular lesions	-	-	-	-	0.0	1.37	0.505	0.001	0.0	1.39	0.707	0.001
Tree-in-bud	-	-	-	-	16.9	3.61	0.307	<0.001	50.8	8.13	0.087	0.305
Organizing pneumonia	62.7	2.68	0.102	0.404	0.0	2.83	0.726	0.001	0.0	1.51	0.959	0.001
Septal thickening	-	-	-	-	12.5	4.57	0.334	<0.001	8.1	5.44	0.365	<0.001
Crazy paving	-	-	-	-	66.7	3.00	0.083	2.145	0.0	0.73	0.696	0.001
Pleural effusion	-	-	-	-	48.7	1.95	0.163	0.228	0.0	2.57	0.463	0.085

**Table A4:** Summary of the results for Egger’s test performed on the main findings reported in the present meta-analysis.

Finding	t	df	Bias (SE)	Intercept (SE)	*p*-Value
Normal findings	−2.64	7	−2.399 (0.910)	0.172	0.034
Bilateral involvement	-	-	-	-	-
Extensive/multifocal	-	-	-	-	-
Ground glass opacities	−1.10	8	−1.957 (1.784)	0.434 (0.941)	0.305
Nodular lesions	−5.52	6	−3.184 (0.577)	1.388 (0.299)	0.002
Organizing pneumonia	−1.37	8	−1.865 (1.358)	0.399 (0.652)	0.207
Septal thickening	−1.19	6	−1.234 (1.036)	0.062 (0.527)	0.279
Tree in bud	−2.75	5	−2.948 (1.071)	0.902 (0.553)	0.040
Crazy paving	-	-	-	-	-
Pleural effusion	−4.32	7	−3.467 (0.803)	1.432 (0.485)	0.004
